# Response to letter to the editor from Thea and Buschard: “Progression from stage 1 to stage 3 type 1 diabetes characterized by hypoglycemia”

**DOI:** 10.1210/jcemcr/luag157

**Published:** 2026-07-02

**Authors:** Jamie L Felton

**Affiliations:** Department of Pediatrics, Center for Diabetes and Metabolic Diseases, Indianapolis, IN 46202, USA; Herman B Wells Center for Pediatric Research, Indiana University School of Medicine, Indianapolis, IN 46202, USA

Dear editor,

We appreciate the thoughtful comments by Thea and Buschard [1] regarding our recent report describing rapid progression from stage 1 to stage 3 type 1 diabetes (T1D) with persistent hypoglycemia [2]. Their proposed mechanism links sulfatide deficiency and antisulfatide antibodies to dysregulated insulin secretion, offering a plausible explanation for both hypoglycemia and accelerated progression in early T1D. We agree that mechanisms underlying hypoglycemia in early T1D remain incompletely understood and that this hypothesis offers an important and compelling framework for further investigation.

At present, direct clinical evidence linking sulfatide deficiency to hypoglycemia in early T1D remains sparse. Reduced sulfatide levels have also been linked to other inflammatory conditions, including multiple sclerosis and lupus nephritis [3, 4], raising questions about disease specificity. In addition, sulfatides exhibit context dependent biologic effects. Su et al [5]. demonstrated that sulfatide act as Toll-like receptor 4/myeloid differentiation factor-2 agonists in mice but antagonists in humans, suggesting they may either promote or suppress inflammation depending on the species and physiologic context.

Similarly, evidence supporting a pathogenic role for antisulfatide antibodies remains inconclusive. These antibodies have been identified across multiple autoimmune conditions, including multiple sclerosis, autoimmune neuropathies, and celiac disease [6-8], suggesting they may reflect generalized immune dysregulation rather than a biomarker specific to rapid T1D progression. Whether individuals with high antisulfatide antibody burdens across autoimmune diseases exhibit increased diabetes risk remains unknown and could point to shared pathogenic mechanisms.

The observations summarized in this letter support the need for further investigation. Identification of antisulfatide antibody patterns in serum samples from longitudinal at-risk cohort studies, together with interrogation of existing omics datasets from these individuals, could help establish whether sulfatide dysregulation precedes rapid progression and identify patients at highest risk of hypoglycemia. It would also be valuable to understand whether sulfatide contributes to the heterogeneity of disease progression among individuals with T1D. For example, were sulfatide levels in the patient presented in our case report significantly lower than those in a peer who progressed less quickly and without hypoglycemia?

The potential dual role for sulfatide in beta cell regulation and immune modulation [5] makes it an attractive therapeutic target. However, timing may be critical. If reduced sulfatide levels contribute to excessive insulin secretion during early disease, restoring sulfatide signaling late in stage 3 could further suppress residual beta cell function rather than preserve it.

In summary, the hypothesis proposed by Thea and Buschard offers an intriguing framework linking sulfatide biology to hypoglycemia and rapid T1D progression. While current evidence remains limited, these observations highlight the need for further mechanistic and longitudinal investigation. A better understanding of sulfatide biology in early T1D may ultimately provide insight into disease heterogeneity and identify novel opportunities for therapeutic intervention.

## Disclosures

J.L.F. has served on advisory boards for Sanofi and has given lectures for MedLearning.
